# *catena*-Poly[[bis­(nitrato-κ^2^ *O*,*O*′)copper(II)]-μ-2,2′-(ethane-1,2-diyldithio)di-1,3,4-thia­diazole-κ^2^ *N* ^4^:*N* ^4′^]

**DOI:** 10.1107/S1600536808041202

**Published:** 2008-12-17

**Authors:** Hua-Ming Huang, Feng-Yang Ju, Jian-Ge Wang, Jian-Hua Qin

**Affiliations:** a College of Chemistry and Chemical Engineering, Luoyang Normal University, Luoyang 471022, People’s Republic of China

## Abstract

In the title compound, [Cu(NO_3_)_2_(C_6_H_6_N_4_S_4_)]_*n*_, the Cu^II^ atom, occupying a crystallographic inversion centre, is six-coordinated by two N atoms of two 2,2′-[1,2-ethane­diyl­bis­(thio)]bis­[1,3,4-thia­diazole] ligands in *trans* positions, and four O atoms from two symmetry-related opposite nitrate anions, which are asymmetrically bonded, resulting in a strong distorted octa­hedral geometry of the central atom. The ethane group is equally disordered over two sites *via* another inversion centre. The bridging bidentate 2,2′-[1,2-ethanediylbis(thio)]bis­[1,3,4-thia­diazole] ligands link the Cu^II^ centres into a one-dimensional chain. The chains are inter­connected *via* inter­molecular S⋯O inter­actions [3.044 (4) and 3.084 (5) Å] and weak C—H⋯O hydrogen bonds, generating a three-dimensional supra­molecular structure.

## Related literature

For related *catena*-poly Cu(II) complexes, see, for example: Wang *et al.* (2008 ▶). For elongated Cu—O bonds see, for example: Lee & Barboiu (2004 ▶); Youngme *et al.* (2007 ▶). For C—H⋯O hydrogen bonds, see: Bhogala *et al.* (2005 ▶).
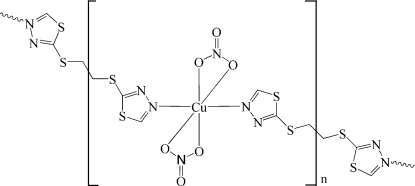


## Experimental

### 

#### Crystal data

[Cu(NO_3_)_2_(C_6_H_6_N_4_S_4_)]
*M* *_r_* = 449.95
Triclinic, 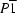

*a* = 5.2143 (8) Å
*b* = 7.0214 (10) Å
*c* = 10.6476 (16) Å
α = 105.144 (2)°
β = 100.000 (2)°
γ = 93.958 (2)°
*V* = 367.88 (9) Å^3^
*Z* = 1
Mo *K*α radiation
μ = 2.09 mm^−1^
*T* = 296 (2) K
0.48 × 0.29 × 0.04 mm

#### Data collection

Bruker SMART CCD area-detector diffractometer
Absorption correction: multi-scan (*SADABS*; Bruker, 1997 ▶) *T*_min_ = 0.432, *T*_max_ = 0.924
1983 measured reflections
1336 independent reflections
1273 reflections with *I* > 2σ(*I*)
*R*_int_ = 0.020

#### Refinement

*R*[*F* ^2^ > 2σ(*F* ^2^)] = 0.058
*wR*(*F* ^2^) = 0.173
*S* = 1.05
1336 reflections
110 parameters
1 restraint
H-atom parameters constrained
Δρ_max_ = 1.86 e Å^−3^
Δρ_min_ = −0.50 e Å^−3^

### 

Data collection: *SMART* (Bruker, 1997 ▶); cell refinement: *SAINT* (Bruker, 1997 ▶); data reduction: *SAINT*; program(s) used to solve structure: *SHELXS97* (Sheldrick, 2008 ▶); program(s) used to refine structure: *SHELX97* (Sheldrick, 2008 ▶); molecular graphics: *SHELXTL* (Sheldrick, 2008 ▶); software used to prepare material for publication: *SHELXTL*.

## Supplementary Material

Crystal structure: contains datablocks I, global. DOI: 10.1107/S1600536808041202/si2137sup1.cif

Structure factors: contains datablocks I. DOI: 10.1107/S1600536808041202/si2137Isup2.hkl

Additional supplementary materials:  crystallographic information; 3D view; checkCIF report

## Figures and Tables

**Table:** 

Cu1—O1	2.588 (4)
Cu1—O3	1.971 (3)
Cu1—N2	2.007 (4)

**Table:** 

O1—Cu1—O3	54.74 (14)
N2—Cu1—O3	89.02 (15)
O1—Cu1—O3^i^	125.26 (14)

Symmetry code: (i) 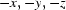.

**Table 2:** Hydrogen-bond geometry (Å, °)

*D*—H⋯*A*	*D*—H	H⋯*A*	*D*⋯*A*	*D*—H⋯*A*
C1—H1*A*⋯O1^ii^	0.93	2.49	3.083 (6)	122

Symmetry code: (ii) 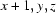.

**Fig. 1.**
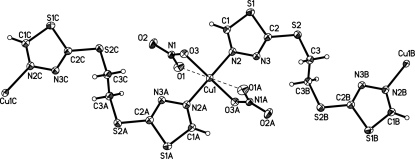
A view of the local coordination of the Cu(II) cation in the title compound. Displacement ellipsoids are drawn at the 30% probability level. The disordered ethane group was omitted for clarity. Symmetry codes: (A) (-x, -y, -z); (B) (1 - x, 1 - y, 1 - z); (C) (-1 + x, -1 + y, -1 + z).

**Fig. 2.**
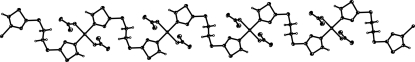
A view of the polymeric chain in the title compound.

**Fig. 3.**
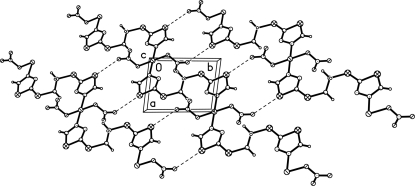
A view of the two-dimensional network, indicating the S···O interactions by dashed lines.

**Fig. 4.**
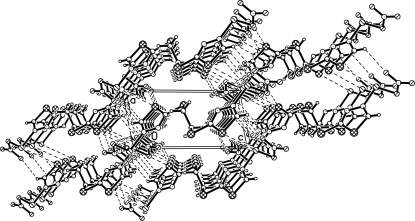
A view of the compound packing down the b axis.

## supplementary crystallographic information

### Comment

As shown in Fig. 1, the copper atom is coordinated by four O atoms from two chelating nitrate anions and two N atoms from two C_i_ symmetry-related 2,2'-[1,2-ethanediylbis(thio)]bis[1,3,4-thiadiazole] ligands. The geometry around the Cu(II) atom appears to be strong distorted octahedral, which is shown with the angle O1—Cu1—O3 = 54.74 (14) °. The nitrate anions are asymmetrically bonded, with Cu1—O3 = 1.971 (3) Å and Cu1—O1 = 2.588 (4) Å (Table 1). Two examples of asymmetrically bonded carboxylate groups show the wide range of short and long Cu(II)–O distances: 1.989 (2) Å and 2.339 (3) Å (Youngme *et al.*, 2007); 1.962 (3) Å and 2.706 Å were reported by Lee & Barboiu (2004).

The ethane group was disordered and the C3 atom was refined on split positions with occupancy (50:50). The 2,2'-[1,2-ethanediylbis(thio)]bis[1,3,4-thiadiazole] ligands adopt a N, N-bidentate bridging mode in *trans* configuration and bridge the copper atoms into one-dimensional infinite chains, with the bridged Cu-Cu distance of 11.2455 (12) Å (Fig. 2). The chains interact with neigboring molecules *via* intermolecular S···O interactions (the shortest distances found: O2···S1 = 3.084 (5) Å and O2···S2 = 3.044 (4) Å, with symmetry codes (1 - *x*, -1 - *y*, -*z*) and (1 - *x*, 1 - *y*, 1 - *z*), respectively, and weak intermolecular C—H···O hydrogen bonds (Bhogala *et al.* (2005), (Table 2). These chains are linked by the S···O interactions into two-dimensional layers (Fig. 3), which are further connected by weak intermolecular C—H···O hydrogen bonds to generate a a three-dimensional supramolecular structure (Fig. 4).

### Experimental

The reaction of 2,2'-[1,2-ethanediyl-bis(thio)]bis(1,3,4- thiadiazole) (0.2 mmol) with Cu(NO_3_)_2_ (0.2 mmol) in MeOH(10 ml) for a few minutes afforded a light blue solid, which was filtered, washed with acetone, and dried on air. The single crystals suitable for X-ray analysis were obtained by slow diffusion of Et_2_O into the acetonitrile solution of the solid.

### Refinement

All hydrogen atoms were positioned geometrically and treated as riding, with C—H = 0.93 Å (CH) and *U*ĩso~(H) = 1.2Ueq(C), with C—H = 0.97 Å (CH2) and *U*ĩso~(H) = 1.2Ueq(C),

### Crystal data

**Table:**

[Cu(NO_3_)_2_(C_6_H_6_N_4_S_4_)]	*Z* = 1
*M**_r_* = 449.95	*F*(000) = 225
Triclinic, *P*1	*D*_x_ = 2.031 Mg m^−^^3^
Hall symbol: -P 1	Mo *K*α radiation, λ = 0.71073 Å
*a* = 5.2143 (8) Å	Cell parameters from 1855 reflections
*b* = 7.0214 (10) Å	θ = 3.0–29.2°
*c* = 10.6476 (16) Å	µ = 2.09 mm^−^^1^
α = 105.144 (2)°	*T* = 296 K
β = 100.000 (2)°	Plate, colorless
γ = 93.958 (2)°	0.48 × 0.29 × 0.04 mm
*V* = 367.88 (9) Å^3^	

### Data collection

**Table:**

Bruker SMART CCD area-detector diffractometer	1336 independent reflections
Radiation source: fine-focus sealed tube	1273 reflections with *I* > 2σ(*I*)
graphite	*R*_int_ = 0.020
phi and ω scans	θ_max_ = 25.5°, θ_min_ = 3.0°
Absorption correction: multi-scan (*SADABS*; Bruker, 1997)	*h* = −6→3
*T*_min_ = 0.432, *T*_max_ = 0.924	*k* = −8→8
1983 measured reflections	*l* = −11→12

### Refinement

**Table:**

Refinement on *F*^2^	Primary atom site location: structure-invariant direct methods
Least-squares matrix: full	Secondary atom site location: difference Fourier map
*R*[*F*^2^ > 2σ(*F*^2^)] = 0.058	Hydrogen site location: inferred from neighbouring sites
*wR*(*F*^2^) = 0.173	H-atom parameters constrained
*S* = 1.05	*w* = 1/[σ^2^(*F*_o_^2^) + (0.1363*P*)^2^ + 0.4692*P*] where *P* = (*F*_o_^2^ + 2*F*_c_^2^)/3
1336 reflections	(Δ/σ)_max_ < 0.001
110 parameters	Δρ_max_ = 1.86 e Å^−^^3^
1 restraint	Δρ_min_ = −0.50 e Å^−^^3^

### Fractional atomic coordinates and isotropic or equivalent isotropic displacement parameters (Å^2^)

**Table:**

	*x*	*y*	*z*	*U*_iso_*/*U*_eq_	Occ. (<1)
C3	0.6301 (17)	0.4878 (11)	0.4823 (9)	0.0366 (14)	0.499 (9)
H3A	0.7602	0.5921	0.5422	0.044*	0.499 (9)
H3B	0.6243	0.4972	0.3926	0.044*	0.499 (9)
C3'	0.4746 (17)	0.4163 (12)	0.5298 (9)	0.0366 (14)	0.501 (9)
H3B'	0.2988	0.3485	0.4917	0.044*	0.501 (9)
H3A'	0.4890	0.4680	0.6249	0.044*	0.501 (9)
Cu1	0.0000	0.0000	0.0000	0.0312 (3)	
S1	0.6997 (3)	−0.12017 (17)	0.26648 (12)	0.0406 (4)	
S2	0.7207 (3)	0.23982 (18)	0.49437 (12)	0.0455 (4)	
O1	−0.2024 (8)	−0.3668 (6)	−0.0576 (4)	0.0546 (10)	
O2	−0.0393 (10)	−0.5352 (6)	−0.2188 (4)	0.0650 (12)	
O3	0.1101 (7)	−0.2255 (5)	−0.1244 (3)	0.0417 (8)	
N1	−0.0491 (8)	−0.3829 (5)	−0.1353 (4)	0.0385 (9)	
N2	0.3011 (8)	−0.0167 (6)	0.1409 (4)	0.0345 (8)	
N3	0.3672 (7)	0.1312 (5)	0.2605 (4)	0.0370 (9)	
C1	0.4554 (9)	−0.1563 (6)	0.1321 (4)	0.0355 (10)	
H1A	0.4330	−0.2663	0.0583	0.043*	
C2	0.5716 (9)	0.0958 (6)	0.3353 (4)	0.0347 (10)	

### Atomic displacement parameters (Å^2^)

**Table:**

	*U*^11^	*U*^22^	*U*^33^	*U*^12^	*U*^13^	*U*^23^
C3	0.036 (4)	0.032 (3)	0.038 (3)	0.005 (2)	0.007 (3)	0.004 (3)
C3'	0.036 (4)	0.032 (3)	0.038 (3)	0.005 (2)	0.007 (3)	0.004 (3)
Cu1	0.0330 (5)	0.0269 (5)	0.0289 (5)	0.0069 (3)	0.0028 (3)	0.0009 (3)
S1	0.0423 (7)	0.0364 (7)	0.0419 (7)	0.0170 (5)	0.0040 (5)	0.0086 (5)
S2	0.0479 (8)	0.0415 (7)	0.0367 (7)	0.0153 (5)	−0.0092 (5)	0.0016 (5)
O1	0.062 (2)	0.049 (2)	0.059 (2)	0.0151 (17)	0.020 (2)	0.0183 (17)
O2	0.088 (3)	0.0380 (19)	0.053 (2)	0.024 (2)	−0.002 (2)	−0.0102 (17)
O3	0.0405 (18)	0.0406 (17)	0.0388 (17)	0.0109 (14)	0.0058 (14)	0.0017 (14)
N1	0.049 (2)	0.0291 (17)	0.0304 (18)	0.0149 (16)	−0.0031 (17)	0.0004 (14)
N2	0.0384 (19)	0.0289 (17)	0.0321 (19)	0.0068 (15)	0.0039 (15)	0.0025 (14)
N3	0.039 (2)	0.0306 (19)	0.035 (2)	0.0102 (17)	−0.0015 (17)	0.0016 (16)
C1	0.039 (2)	0.032 (2)	0.033 (2)	0.0098 (18)	0.0062 (18)	0.0034 (17)
C2	0.037 (2)	0.0291 (19)	0.035 (2)	0.0078 (18)	0.0048 (19)	0.0054 (17)

### Geometric parameters (Å, °)

**Table:**

C3—C3^i^	1.479 (17)	Cu1—N2^ii^	2.007 (4)
C3—S2	1.866 (7)	S1—C1	1.693 (5)
C3—H3A	0.9700	S1—C2	1.735 (4)
C3—H3B	0.9700	S2—C2	1.743 (4)
C3'—C3'^i^	1.504 (17)	O1—N1	1.237 (6)
C3'—S2	1.863 (7)	O2—N1	1.209 (5)
C3'—H3B'	0.9700	O3—N1	1.303 (5)
C3'—H3A'	0.9700	N2—C1	1.305 (6)
Cu1—O1	2.588 (4)	N2—N3	1.389 (5)
Cu1—O3^ii^	1.971 (3)	N3—C2	1.295 (6)
Cu1—O3	1.971 (3)	C1—H1A	0.9300
Cu1—N2	2.007 (4)		
			
C3^i^—C3—S2	108.9 (7)	C2—S2—C3'	100.6 (3)
C3^i^—C3—H3A	109.9	C2—S2—C3	99.5 (3)
S2—C3—H3A	109.9	N1—O3—Cu1	107.4 (2)
C3^i^—C3—H3B	109.9	O2—N1—O1	123.7 (5)
S2—C3—H3B	109.9	O2—N1—O3	119.2 (4)
H3A—C3—H3B	108.3	O1—N1—O3	117.1 (4)
C3'^i^—C3'—S2	108.5 (7)	O1—Cu1—O3	54.74 (14)
C3'^i^—C3'—H3B'	110.0	N2—Cu1—O3	89.02 (15)
S2—C3'—H3B'	110.0	O1—Cu1—O3^ii^	125.26 (14)
C3'^i^—C3'—H3A'	110.0	C1—N2—N3	113.3 (4)
S2—C3'—H3A'	110.0	C1—N2—Cu1	126.2 (3)
H3B'—C3'—H3A'	108.4	N3—N2—Cu1	120.5 (3)
O3^ii^—Cu1—O3	180.0	C2—N3—N2	110.7 (4)
O3^ii^—Cu1—N2	90.98 (15)	N2—C1—S1	114.2 (3)
O3—Cu1—N2	89.02 (15)	N2—C1—H1A	122.9
O3^ii^—Cu1—N2^ii^	89.02 (15)	S1—C1—H1A	122.9
O3—Cu1—N2^ii^	90.98 (15)	N3—C2—S1	114.8 (3)
N2—Cu1—N2^ii^	180.0	N3—C2—S2	126.5 (3)
C1—S1—C2	87.1 (2)	S1—C2—S2	118.7 (3)
			
C3'^i^—C3'—S2—C2	−83.9 (9)	Cu1—N2—N3—C2	−177.8 (3)
C3'^i^—C3'—S2—C3	7.8 (7)	N3—N2—C1—S1	−1.2 (5)
C3^i^—C3—S2—C2	87.1 (9)	Cu1—N2—C1—S1	177.3 (2)
C3^i^—C3—S2—C3'	−7.9 (7)	C2—S1—C1—N2	0.9 (4)
N2—Cu1—O3—N1	103.2 (3)	N2—N3—C2—S1	−0.1 (5)
N2^ii^—Cu1—O3—N1	−76.8 (3)	N2—N3—C2—S2	−179.2 (3)
Cu1—O3—N1—O2	171.2 (4)	C1—S1—C2—N3	−0.4 (4)
Cu1—O3—N1—O1	−9.2 (4)	C1—S1—C2—S2	178.7 (3)
O3^ii^—Cu1—N2—C1	171.9 (4)	C3'—S2—C2—N3	9.6 (5)
O3—Cu1—N2—C1	−8.1 (4)	C3—S2—C2—N3	−27.8 (5)
O3^ii^—Cu1—N2—N3	−9.7 (3)	C3'—S2—C2—S1	−169.5 (4)
O3—Cu1—N2—N3	170.3 (3)	C3—S2—C2—S1	153.1 (3)
C1—N2—N3—C2	0.8 (6)		

Symmetry codes: (i) −*x*+1, −*y*+1, −*z*+1; (ii) −*x*, −*y*, −*z*.

### Hydrogen-bond geometry (Å, °)

**Table:**

*D*—H···*A*	*D*—H	H···*A*	*D*···*A*	*D*—H···*A*
C1—H1A···O1^iii^	0.93	2.49	3.083 (6)	122

Symmetry codes: (iii) *x*+1, *y*, *z*.
